# Inhibitory effect of ethanol extract of *Ocimum sanctum* on osteopontin mediated metastasis of NCI-H460 non-small cell lung cancer cells

**DOI:** 10.1186/1472-6882-14-419

**Published:** 2014-10-27

**Authors:** Tae-kyung Kwak, Eun Jung Sohn, Sunhee Kim, Gunho Won, Jeong-Un Choi, Kwon Jeong, Myoungseok Jeong, Oh Sung Kwon, Sung-Hoon Kim

**Affiliations:** College of Oriental Medicine, Kyung Hee University, Hoegidong, Dongdaemungu, Seoul, 130-701 Republic of Korea

**Keywords:** EEOS, Metastasis, Osteopontin, uPA, uPAR, PI3K

## Abstract

**Background:**

Osteopontin (OPN) is one of important molecular targets in cancer progression, metastasis as a calcium-binding, extracellular-matrix-associated protein of the small integrin-binding ligand and, N-linked glycoprotein. In the present study, anti-metastatic mechanism of ethanol extracts of *Ocimum sanctum* (EEOS) was elucidated on OPN enhanced metastasis in NCI-H460 non- small cell lung cancer cells.

**Methods:**

Cell viability was measured by MTT assay. Adhesion and invasion assays were carried out to see that EEOS inhibited cell adhesion and invasion in OPN treated and non-treated NCI-H 460 cells. RT-PCR was used to determine the mRNA levels of uPA, uPAR, and EGFR.

**Results:**

EEOS significantly inhibited cell adhesion and invasion in OPN treated and non treated NCI-H460 cells, though EEOS did not show any toxicity up to 200 μg/ml. EEOS effectively attenuated the expression of OPN and CD44 and also OPN activated the expression of CD44 in NCI-H460 cells. In addition, EEOS effectively suppressed the expression of phosphatidylinositide 3-kinases (PI3K) and cyclooxygenase 2 (COX-2) and the phosphorylation of Akt at protein level in OPN treated NCI-H460 cells. Also, EEOS significantly attenuated the expression of urokinase plasminogen activator (uPA), its receptor (uPAR) and epidermal growth factor receptor (EGFR) at mRNA level and reduced vascular endothelial growth factor (VEGF) production and MMP-9 activity in OPN treated NCI-H460 cells. Furthermore, PI3K/Akt inhibitor LY294002 enhanced anti-metastatic potential of EEOS to attenuate the expression of uPA and MMP-9 in OPN treated NCI-H 460 cells.

**Conclusion:**

Overall, our findings suggest that anti-metastatic mechanism of EEOS is mediated by inhibition of PI3K/Akt in OPN treated NCI-H460 non-small cell lung cancer cells.

## Background

Metastasis, one of malignant tumor features, is controlled by multi-step processes including tumor angiogenesis, tumor invasion, and establishment metastatic foci at the secondary site via various molecular targets [1, 2].

Osteopontin (OPN) is a secreted phosphorylated glycoprotein that is closely involved in inflammation [3, 4], kidney stone formation [5, 6], tumor migration and metastasis [7–9]. Also, it was well documented that activation of matrix metalloproteinase (MMP)-2 and -9 as a proteolytic enzyme in extra cellular matrix (ECM) [10] and urokinase plasminogen activator (uPA) [11, 12] and its receptor (uPAR) [13] is closely involved in metastasis and cancer invasion. In addition, P13K/Akt pathway was known to play a key role in cancer metastasis in cholangiocarcinoma [14], liver cancer [15], colorectal cancer [16], lung cancer [17], esophageal cancer [18] and ovarian cancer [19].

Recently many medicinal herbs [20, 21] and phytochemicals [15, 22–24] are attractive with anti-metastatic potential with low toxicity. *Ocimum sanctum* Linne, commonly known as ‘Holy basil’ , [25] was known to have multi-biological effects on immunomodulation, anti-ulcer, anti-inflammation and anti-carcinogenesis [26–29]. Furthermore, the ethanol (70%) extract of *O. sanctum* possess anti-hyperglycaemic action [30] and anti-fatigue property in rats [31].

Though our group previously reported ethanol extract (95%) of OS (EEOS) exerts anti-metastatic activity via inhibition of MMP-9 and enhancement of antioxidant enzymes [32], the underlying anti-metastatic mechanism of EEOS still remains unclear. Thus, in the present study, anti-metastatic mechanism of EEOS was elucidated via cell viability assay, cell adhesion and invasion assays, ELISA for MMP-9, RT-PCR for uPA, uPAR and EGFR, Western blotting for osteopontin (OPN), and CD44, ELISA for VEGF and PI3K/Akt inhibitor LY294002 study in OPN treated NCI-H460 non-small cell lung cancer cells.

## Methods

### Preparation of EEOS

*O.sanctum Linne* collected in Chennai, India was identified by Dr. Namin Baek, a professor and pharmacognosist at Kyung hee University, Korea and stored at the Cancer Preventive Material Development Research Center (CPMDRC), Kyung hee University at Korea. The extraction of *O. sanctum Linne* was carried out according to standard protocols as described previously [32]. To extract the EEOS, 95% ethanol was added in the leaves of *O. sanctum Linne* (3 kg) and incubated for 3 days at room temperature. A rotary evaporator (Eyela, Tokyo, Japan) was used to concentrate and freeze-dried to obtain 570 g (yield = 19%) of ethanol extract of *O. sanctum* (EEOS).

### Cell culture

NCI-H460 non-small cell lung cancer cells (HTB-177™) were purchased from American Type Culture Collection (ATCC) and were cultured in RPMI1640 medium (Invitrogen, Carlsbad, CA, USA) supplemented with 10% FBS, 2 mM L-glutamine, and 100 units/ml antibiotic-antimycotics.

### Cytotoxicity assay

The cell viability was assessed by MTT assay (Sigma Chemical Co., St. Louis, MO). NCI-H460 cells were seeded at 1 × 10^4^ cells/well of 96-well flat bottom plate and treated with various concentrations of EEOS (0, 6, 12.5, 25, 50, 100, and 200 μg/ml) for 24 h. MTT reagent was added to each well and incubated for 4 h at 37°C. Formazen crystals were dissolved by addition of dimethyl sulfoxide (DMSO) solution. The absorbance of each well was determined using the microplate reader (Molecular Devices Co., Sunnyvale, CA, USA) at 570 nm. Cell viability of H460 cells was calculated as a percentage of viable cells in EEOS treated group versus control group by a following equation. Cell viability (%) = [OD (EEOS) - OD (Blank)] / [ OD (Control) – OD (Blank)] × 100.

### Adhesion assay

Adhesion assay was performed as previously described [33]. Each well of 96-well plate was coated with Matrigel (11 mg/ml of stock solution) or 0.1% gelatin and incubated for 2 h. The plates were washed and incubated for 1 h with 0.1% bovine serum albumin (BSA) to block unbounded surface. Prior to addition of the cells to each well, OPN treated NCI-H 460 cells (1 × 10^6^) were pre-incubated with EEOS (0, 100 and 200 μg/ml) for 20 min at 37°C. A tetrazolium salt, 2,3-bis[2-methyloxy-4-nitro-5-sulfophenyl]-2H-tetrazolium-5-carboxanilide (XTT) working solution was prepared just prior to culture application by mixing 1 ml of XTT stock solution (1 mg/ml in phosphate buffered saline (PBS)) with 10 μl of phenazine methosulphate (PMS) (1.53 mg/ml in PBS). After incubation at 37°C in a humidified incubator for 24 h, a 50 μl of XTT working solution was added to each well. Cells were incubated at 37°C for 2 h and the optical density was measured by microplate reader (Sunrise, TECAN, Männedorf, Switzerland) at 630 nm.

### Invasion assay

Boyden chamber (Neuro Probe Inc., Gaithersburg, MD, USA) was used to evaluate the spontaneous invasion of NCI-H460 cells as previously described [34]. The method is based on the passage of cells across porous filters separating the upper and lower wells of the migration chamber using OPN (50 nM) treated NCI-H 460 cells. Polyvinyl-pyrrolidone-free polycarbonate filters (8 μm pore size) were used in this experiment. The filters were coated with the reconstituted basement membrane Matrigel (50 μg/filter). NCI-H460 cells were pre-cultured in FBS free-Dulbecco’s Modified Eagle Medium (DMEM) in the absence or presence of EEOS for 7 h and then were loaded onto the upper compartment of the Boyden chamber with various concentrations of EEOS (100 and 200 μg/ml). FBS free Minimum Essential Medium (MEM) containing 0.1% BSA was placed in the lower compartment of the Boyden chamber. The migration was allowed to occur in the absence (control condition) or presence of EEOS in the medium of the upper and the lower compartment of the migration chamber. The chamber was incubated at 37°C for 7 h and the filters were removed and fixed in methanol. Non-migrated cells on the upper surface of the filter were removed with a cotton swab, while migrated cells, adherent on the lower filter surface, were stained with Diff-Quick (Mertz-Dade AG, Dade International, Milan, Italy) and counted under a light microscope (×400 in 10 random fields) per each well. Each experiment was performed in triplicates. Migration values were expressed as means ± S.D. of the number of migrated cells × 100%/total cells counted on the lower surface of filter.

### Western blotting

Whole cell lysates from OPN (50 nM) treated cells exposed to EEOS for 24 h were prepared using lysis buffer (50 mM Tris–HCl, pH 7.4, 300 mM NaCl, 0.5% Triton X-100, 5 mM EDTA, 1 mM Na_3_VO_4_, 1 mM NaF, 10 μg/ml aprotinin, 10 μg/ml leupeptin, 10 μg/ml pepstatin, 10 mM iodoacetamide, 2 mM PMSF). Nuclear protein extracts and cytoplasmic protein extracts were collected by using NE-PER nuclear and cytoplasmic extraction reagents (Thermo scientific, Rockford, IL). The protein contents were measured by using a Bio-Rad DC protein assay kit II (Bio-Rad, Hercules, CA), separated on 10% tris-glycin gels, and electrotransferred onto a Hybond ECL transfer membrane with transfer buffer (25 mM Tris, 250 mM glycine, 20% methanol). The membranes were blocked in 5% nonfat dry milk in TBS buffer containing 0.1% Tween 20 (TBST) and immunoblotted with antibodies of PI3K, p-AKT, OPN, CD44 (Cell signaling, USA).

### *ELISA*for VEGF & MMP-9 activity

The level of VEGF in NCI-H460 cells was measured with a commercially available ELISA kit (R&D systems, Minneapolis, MN). Briefly, the cells were starved for 6 h in M199 containing 5% FBS and then treated with bFGF (50 ng/ml) containing EEOS (50, 100 and 200 μg). After 48 h incubation, the supernatant was individually collected and measured by ELISA kit.

MMP-9 enzymatic activities were assayed by ELISA Kit for MMP-9 (Invitrogen, Camarillo, CA, USA) [35]. NCI-H460 cells plated on 6-well plates were grown to 90% confluence in 2 ml of growth medium in the presence of OPN (50 nM). The cells were maintained in serum-free media and treated with various concentrations of EEOS for 24 h. Conditioned medium was collected and analyzed for the activity of MMP-9 using Human MMP-9 ELISA kit, according to the manufacture’s protocol. Also, PI3K/AKT inhibitor LY294002 study was performed for MMP-9 activity affected by EEOS in OPN treated NCI-H460 cells.

### RT-PCR analysis

Total RNA from OPN (50 nM) treated cells exposed to EEOS for 24 h was isolated using the Trizol reagent according to the manufacturer’s instructions (Invitrogen, Carsbad, CA). One microgram of total RNA was converted to cDNA by Superscript reverse transcriptase and amplified by Platinum *Taq* polymerase using Superscript One Step RT-PCR kit (Invitrogen, Carsbad, CA, USA). Primers were synthesized by Bioneer (Daejeon, Korea) with the following sequences: forward 5′-ACATTCACTGGTGCAACTGC-3′ and reverse 3′-CAAGCGTGTCAGCGCTGTAG-5′ for uPA*;* forward 5′-ATCAGACATGAGCTGTGAGAGG-3′ and reverse 5′-ACTACGGCTCTGAAGTCACCAC-3′ for uPAR; forward 5′-ATGCCCG-CATTAGCTCTTAG-3′ and reverse 5′-GCAACTTCCCAAAATGTGCC-3′ for EGFR; forward 5′-TCACCATCTTCCAGGAGCGA-3′; reverse, 5′-CACAATGCCGAAGTGGTCGT-3′ for GAPDH primers. PCR conditions comprised an initial step at 50°C for 30 min, 94°C for 2 min, followed by 30 cycles at 94°C for 15 s, 55°C for 30 s and 72°C for 1 min, and a final step at 72°C for 10 min. The amplified products were separated on 2% agarose gel. Also, PI3K/Akt inhibitor LY294002 study was performed for uPA affected by EEOS in OPN treated NCI-H460 cells.

### Statistical analysis

All data were presented as means ± standard deviation (S.D.). A one-way ANOVA was used for comparison of multiple groups. Student’s t-test was used for comparison of two groups. Statistical difference was set at *p* values of <0.05 between control and EEOS-treated groups.

## Results

### Effect of EEOS on the viability of NCI-H460 cells

**Figure 1:**
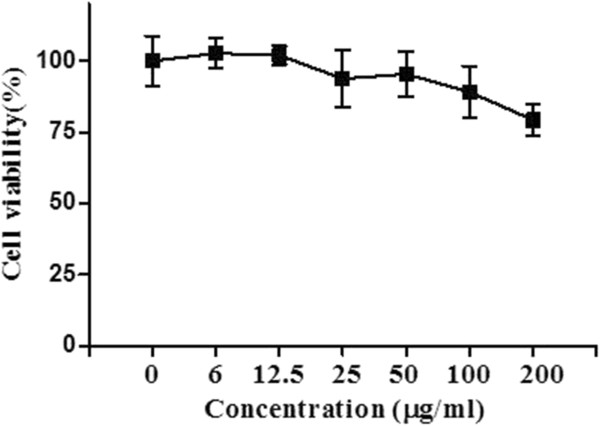
**The cytotoxicity of EEOS on NCI-H460.** Cells were treated with 0, 6, 12.5, 25, 50, 100 or 200 μg/ml of EEOS for 24 h. Cell viability was determined by the MTT assay. Data represent means ± S.D. The cytotoxicity of EEOS was weak up to 200 μg/ml in NCI-H460 cells.

To evaluate cytotoxic effect of EEOS, MTT assay was performed. NCI-H460 cells were plated in 96-well plate and treated with various concentrations (0, 6, 12.5, 25, 50, 100, and 200 μg/ml) of EEOS for 24 h. As shown in Figure 1, the cytotoxicity of EEOS was weak up to 200 μg/ml in NCI-H460 cells. Thus, the next metastasis related experiments were carried out at the concentrations below 200 μg/ml.

### Effects of EEOS on the adhesion and invasion of NCI-H460 cells

**Figure 2:**
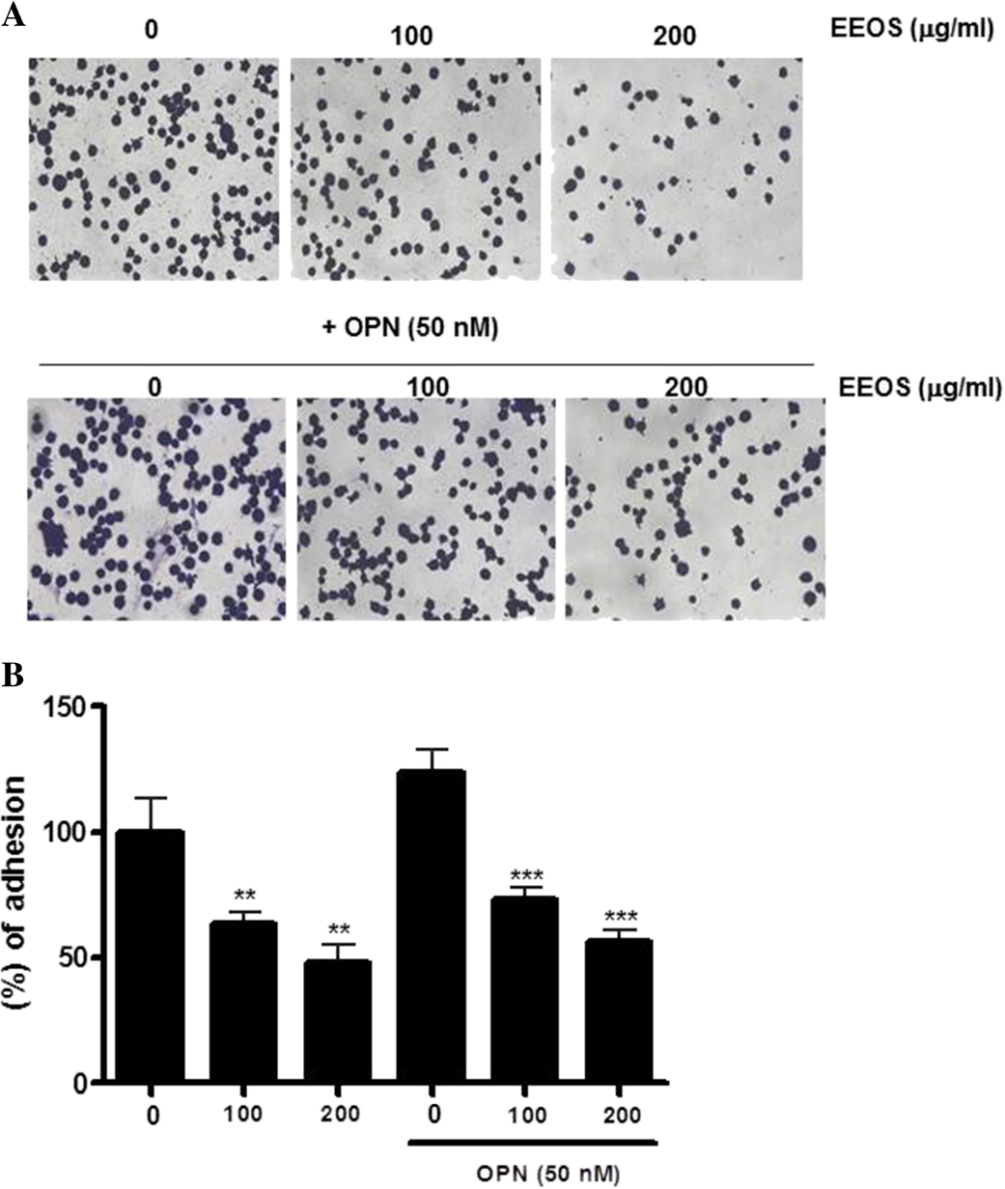
**Inhibitory effect of EEOS on adhesion ability of NCI-H460 cells mediated by osteopontin.** Effect of EEOS in the absence or presence of OPN (50 nM) on the adhesion to Matrigel coated plate after 20 min exposure. Attached cells were photographed (×200) after crystal violet staining **(A)** and were quantified **(B)**. The values represent means ± S.D. of 3 different experiments performed in triplicates. ** , *p* < 0.01, ***, *p* < 0.001, vs untreated control.

**Figure 3:**
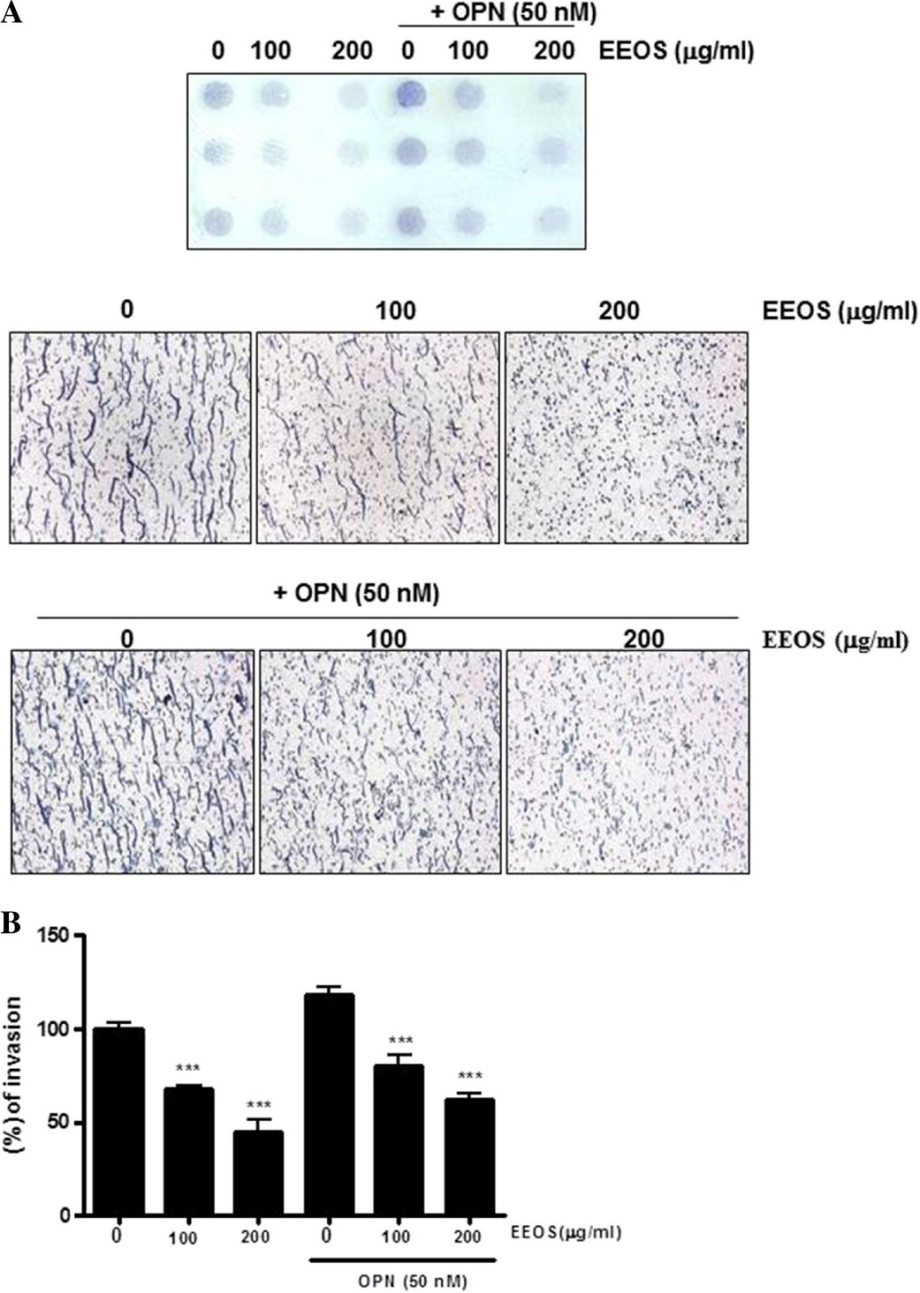
**Inhibitory effect of EEOS on invasion ability of NCI-H460 cells mediated by osteopontin.** Effect of EEOS in the absence or presence of OPN (50 nM) on the invasion ability of NCI-H460 cells. The cells pretreated for 7 h with or without EEOS (0, 100 or 200 μg/ml), were placed in the upper well of the migration chamber and incubated at 37°C for 6 h. The filter was then removed and fixed in methanol. At the end of incubation, images of a number of invaded cells were taken under a light microscope per each field **(A)** and finally quantified **(B)**. *Statistically significant value compared with control data (**p < 0.01).

The first step of metastasis is the adhesion of cancer cells to extracellular matrix (ECM) [36]. To evaluate the adhesive ability of NCI-H460 cells, the cells treated with or without EEOS were added onto Matrigel coated plates. As shown in Figure 2, EEOS significantly inhibited the number of cell adhesion from 100 μg/ml in untreated or OPN treated NCI-H460 cells compared to untreated control. Similarly, EEOS significantly suppressed the number of invaded cells by invasion assay using Boyden-chamber compared to untreated control in untreated or OPN treated NCI-H460 cells (Figure 3).

### Effects of EEOS on metastasis related molecules in OPN treated or untreated NCI-H460 cells

**Figure 4:**
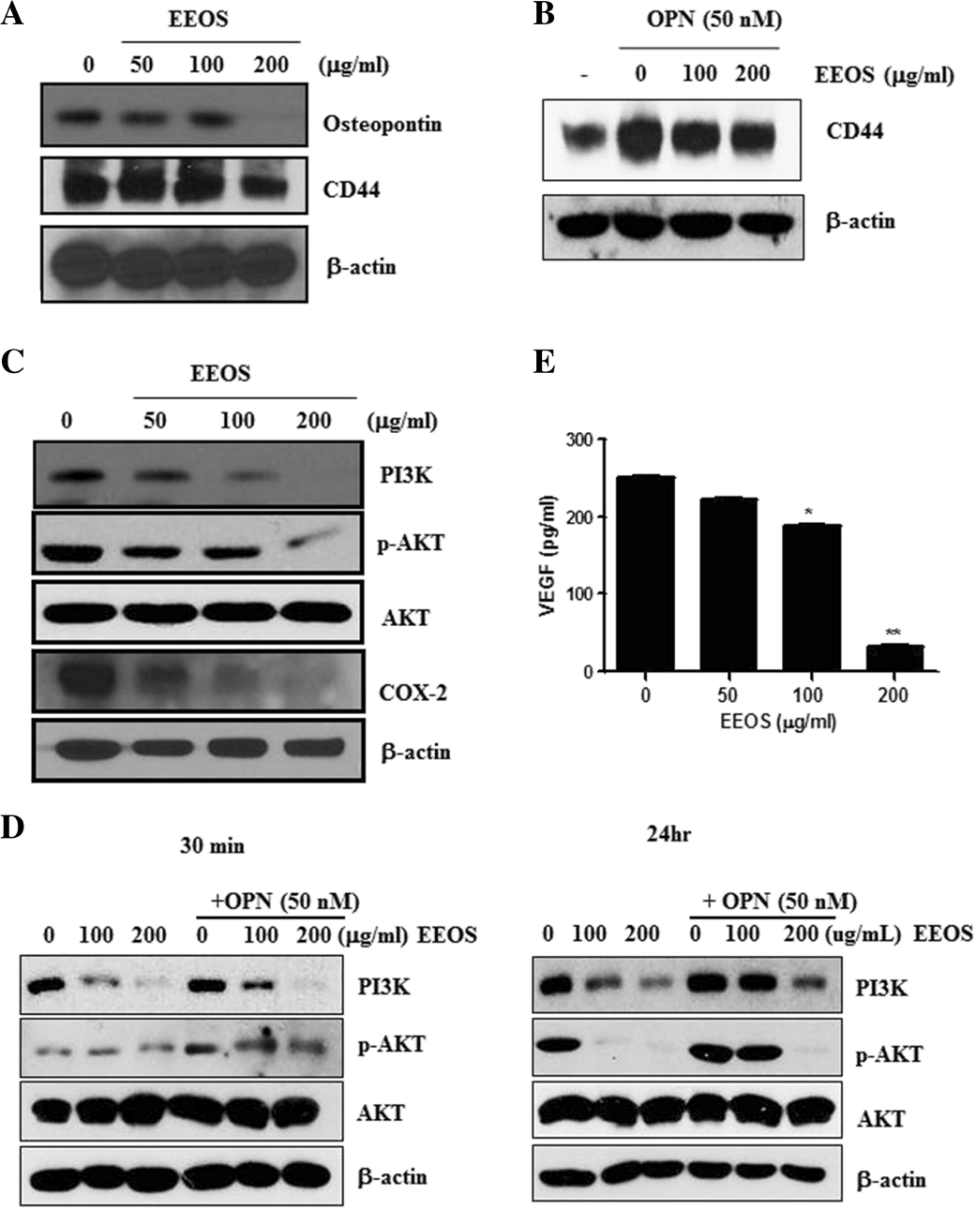
**Effects of EEOS on downregulation of OPN and CD44 and suppression of PI3K/Akt, COX-2 and VEGF in OPN treated NCI-H460. (A)** Effect of EEOS on expression of osteopontin or CD44 in NCI-H460 cells exposed to EEOS for 24 h and Western blotting was performed with antibodies of OPN and CD44. **(B)** Effect of EEOS with treatment of OPN on expression of CD44 in NCI-H460 cells. Western blotting was performed with antibodies of CD44. **(C)** Effect of EEOS on expression of PI3K, COX-2 and the phosphorylation of Akt in NCI-H460 cells. **(D)** Effect of EEOS with treatment of OPN for 30 min or 24 h on expression of PI3K, and the phosphorylation of Akt in OPN treated NCI-H460 cells. **(E)** Effect of EEOS on VEGF production in NCI- H460 cells. The level of VEGF in NCI-H460 cells was measured by ELISA. *P < 0.05, **P < 0.01, versus control.

OPN [9, 37] and CD44 [38, 39] are closely involved in metastasis process. Western blotting revealed that EEOS downregulated the expression of OPN and CD44 in NCI-H460 cells (Figure 4A) and also suppressed the expression of CD44 in OPN treated NCI-H460 cells (Figure 4B).

Our study showed that EEOS attenuated the expression of PI3K and COX-2 and the phosphorylation of Akt at protein level in OPN treated NCI-H460 cells (Figure 4C). Also, EEOS attenuated the expression of PI3K and the phosphorylation of Akt at protein level in OPN treated NCI-H460 cells (Figure 4D).

It was well known that angiogenic biomarker VEGF plays a key role in metastasis [40, 41]. ELISA showed that EEOS significantly reduced VEGF production in NCI-H460 cells (Figure 4E).

**Figure 5:**
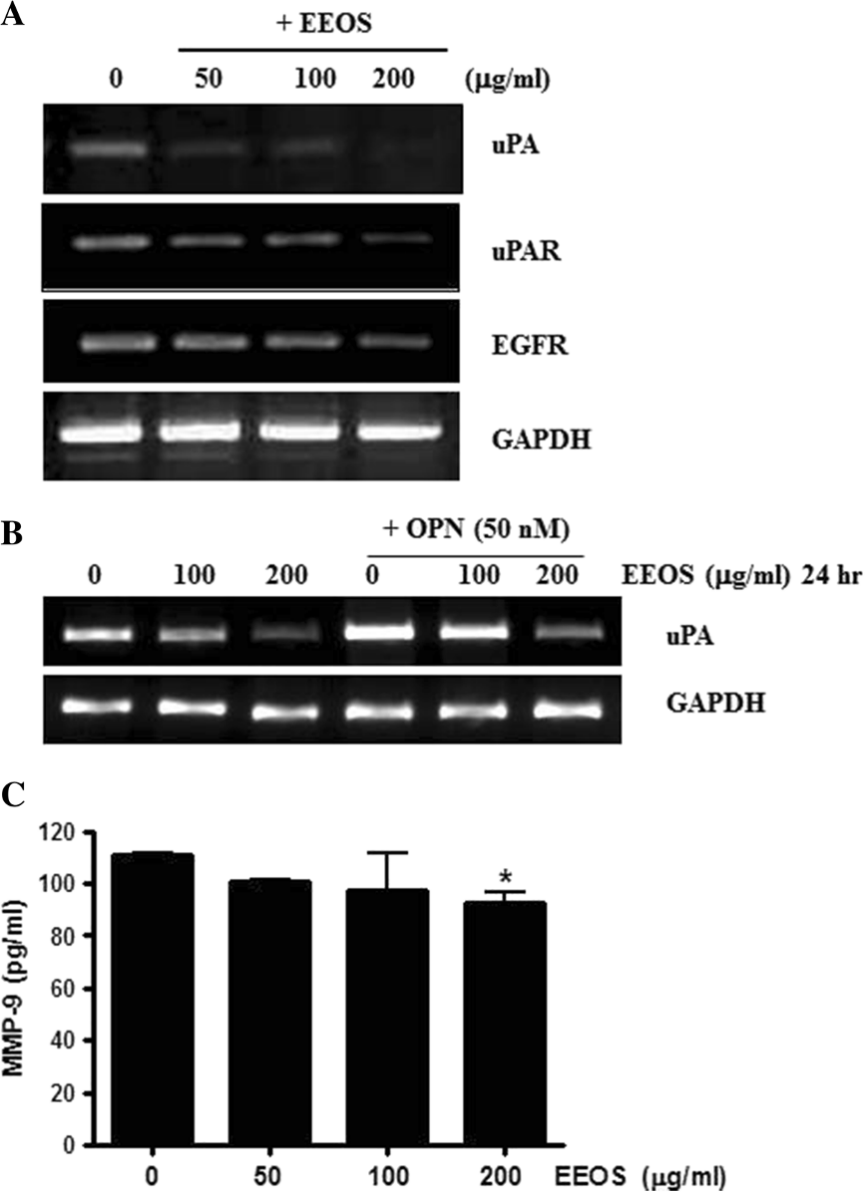
**Effect EEOS on the expression of uPA, uPAR and EGFR in NCI-H460 cells. (A)** RT-PCR analysis showed that EEOS attenuated the expression of uPA, uPAR and EGFR in NCI-H460 cells. **(B)** Effect EEOS on the mRNA expression of uPA in OPN treated NCI-H460 cells. **(C)** Effect EEOS on MMP-9 activity in NCI-H460 cells by ELISA.

**Figure 6:**
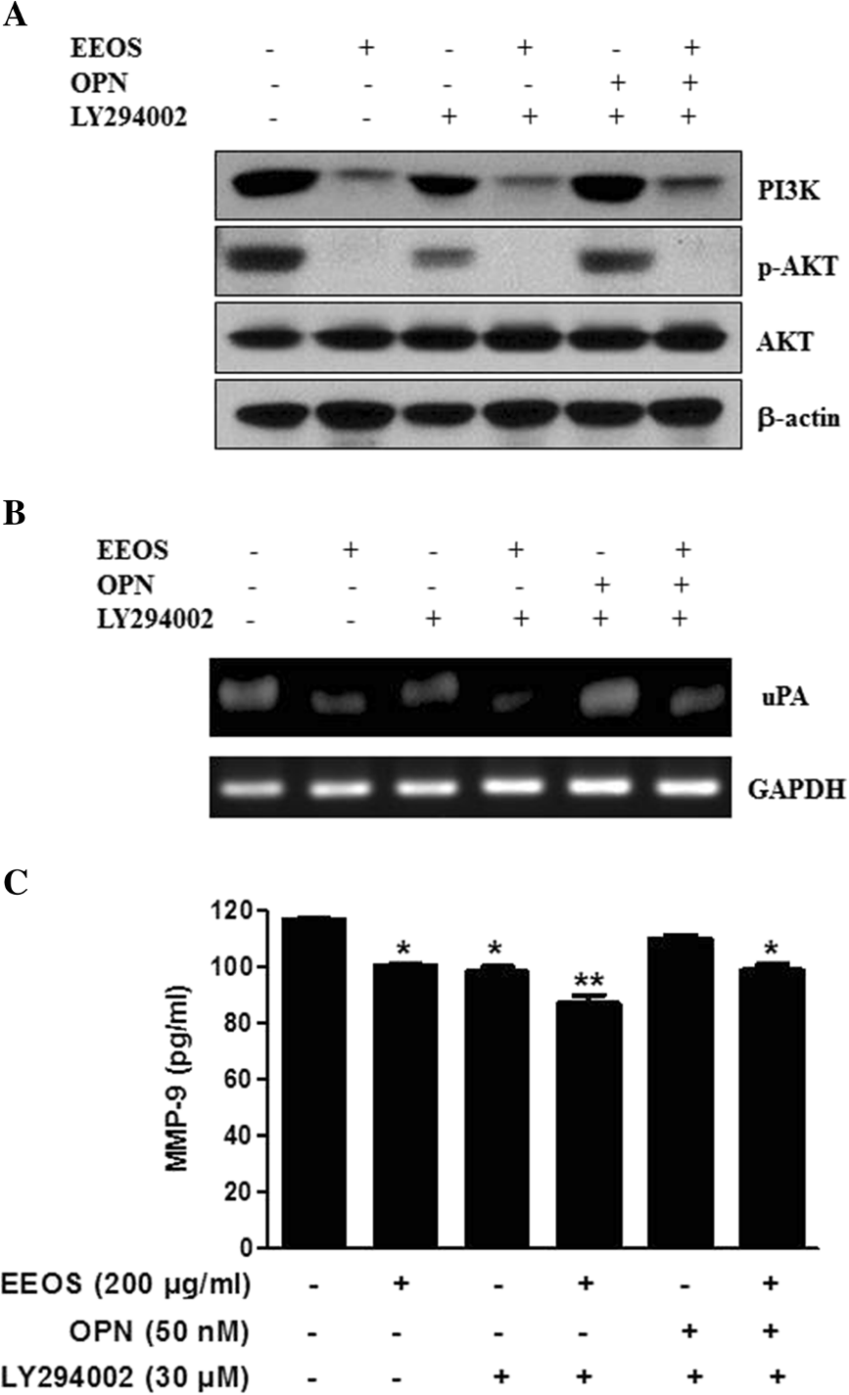
**Effect of PI3K inhibitor LY294002 on PI3K signaling and MMP-9 activity in OPN treated NCI-H460 cells. (A)** Effect of EEOS and LY294002 on PI3K/Akt signaling in OPN treated NCI-H460. **(B)** Effect of EEOS and LY294002 on uPA expression in OPN treated NCI-H460 by RT-PCR. **(C)** Effect of EEOS and LY294002 on MMP-9 activity in OPN treated NCI-H460 by ELISA. Values represent means ± S.D. *P < 0.05, **P < 0.01, versus untreated control.

There are evidences that uPA [12, 42], uPAR [13] and EGFR [43, 44] are closely involved in metastasis. In the current study, RT-PCR analysis exhibited that EEOS attenuated the expression of uPA, uPAR and EGFR in NCI-H460 cells (Figure 5A). Additionally, EEOS attenuated the expression of uPA in OPN treated NCI-H460 cells (Figure 5B). ELISA revealed that EEOS significantly reduced MMP-9 activity in NCI-H460 cells (Figure 5C).Also, PI3K/Akt inhibitor LY294002 enhanced anti-metastatic potential of EEOS to attenuate the expression of uPA and MMP-9 in OPN treated NCI-H 460 cells (Figure 6). EEOS and LY294002 attenuated the expression of PI3K/Akt signaling in OPN treated NCI-H460 (Figure 6A). EEOS and LY294002 attenuated the mRNA expression of uPA (Figure 6B) as well as MMP-9 activity in OPN treated NCI-H460 (Figure 6C).

## Discussion

Though modern medicine has contributed to the treatment of cancers by surgery, chemotherapy and radiotherapy for years, metastasis frequently shown in the patients with malignant neoplasms is still the leading cause of death in cancer patients [45, 46].

Metastasis processes are closely associated with tumor cell dissociation, arrest in small vessels, adhesion to endothelial cells, extravasation, neovascularization, invasion of the target organ, and proliferation [1, 2]. Non-small cell lung carcinomas (NSCLCs), one of epithelial lung cancers, are relatively insensitive to chemotherapy compared with small cell lung carcinoma. A549 and NCI-H460 cells are highly metastatic NSCLCs with p53 wild type [47]. Thus, our study focused the underlying anti-metastatic mechanism of EEOS in OPN treated NCI-H460 NSCLCs, since OPN plays a critical role in metastasis [7, 37].

Adhesion and invasion assays showed that EEOS significantly inhibited cell adhesion and invasion in OPN treated and non treated NCI-H 460 cells, implying that EEOS can suppress OPN mediated metastasis in NSCLCs. There are evidences that OPN and CD 44 play critical roles in metastatic processes [7, 37, 39]. Western blotting revealed that EEOS effectively attenuated the expression of OPN and CD44 and also OPN activated CD44 at nontoxic concentrations in NCI-H460 cells.

It was well documented that phosphatidylinositide 3-kinases (PI3K)/Akt [48, 49] and cyclooxygenase 2 (COX-2) [50, 51] are closely associated with metastasis. Here, EEOS effectively suppressed the expression of PI3K and COX-2 and the phosphorylation of Akt at protein level in OPN treated NCI-H460 cells, indicating that inhibition of PI3K/Akt and COX-2 pathway mediates anti-metastatic effect of EEOS in NCI-H460 cells.

MMPs play important roles in tumor metastasis [52, 53]. As expected, EEOS significantly attenuated the expression of uPA, its receptor uPAR and EGFR in NCI-H460 cells by RT-PCR, reduced VEGF production by ELISA and suppressed MMP-9 expression by ELISA in NCI-H460 cells, implying the involvement of uPA/MMP-9/VEGF signaling. Conversely, PI3K/Akt inhibitor LY294002 enhanced anti-metastatic potential of EEOS to attenuate the expression of uPA and MMP-9 in OPN treated NCI-H 460 cells, demonstrating that inhibition of PI3K/Akt signaling pathway mediates anti-metastatic activity of EEOS in NCI-H 460 cells.

## Conclusions

In summary, EEOS significantly inhibited cell adhesion and invasion at nontoxic concentrations, attenuated the expression of OPN and CD44 and also OPN activated CD44 in NCI-H460 cells. In addition, EEOS effectively suppressed PI3K/Akt, COX-2, uPA, uPAR, MMP-9, VEGF and EGFR in OPN treated NCI-H460 cells. Conversely, PI3K/Akt inhibitor LY294002 enhanced anti-metastatic potential of EEOS to attenuate the expression of uPA and MMP-9 in OPN treated NCI-H 460 cells. Taken together, these findings suggest that inhibition of PI3K/Akt mediates OPN enhanced metastasis in *Ocimum sanctum* treated NCI-H460 non- small cell lung cancer cells.
